# Involvement of tryptophan hydroxylase 2 gene polymorphisms in susceptibility to tic disorder in Chinese Han population

**DOI:** 10.1186/1744-9081-9-6

**Published:** 2013-01-29

**Authors:** Ping Zheng, Erzhen Li, Jianhua Wang, Xiaodai Cui, Liwen Wang

**Affiliations:** 1 Department of Neurological medicine, Capital Institute of Pediatrics, Beijing, 100020, China; 2 Department of Biotechnology, Capital Institute of Pediatrics, Beijing, 100020, China; 3 Internal Medicine Laborary, Capital Institute of Pediatrics, Beijing, 100020, China

**Keywords:** Tic disorder, Tryptophan hydroxylase 2, Single nucleotide polymorphisms

## Abstract

**Background:**

Tryptophan hydroxylase-2 (TPH2) is a potential candidate gene for screening tic disorder (TD).

**Methods:**

A case–control study was performed to examine the association between the TPH2 gene and TD. The Sequenom® Mass ARRAY iPLEX GOLD System was used to genotype two single nucleotide polymorphisms (SNPs) of the TPH2 gene in 149 TD children and in 125 normal controls.

**Results:**

For rs4565946, individuals with the TT genotype showed a significantly higher risk of TD than those with TC plus CC genotypes [odds ratio (OR) =3.077, 95% confidence interval (CI): 1.273–7.437; *P* = 0.009], as did male TD children with the TT genotype (OR = 3.228, 95% CI: 1.153–9.040; *P* = 0.020). The G allele of rs4570625 was significantly more frequent in TD children with higher levels of tic symptoms (Yale Global Tic Severity Scale, YGTSS) than those in controls among the male children (OR = 1.684, 95%: 1.097–2.583; *P* = 0.017]. TD children with severe tic symptoms had significantly higher frequencies of rs4546946 TT genotype than did normal controls in boys (OR = 3.292, 95% CI: 1.139–9.513; *P* = 0.022). We also found that genotype distributions of both SNPs were different between the Asian and European populations.

**Conclusions:**

Our results indicated that the TT genotype of rs4565946 is a potential genetic risk factor for TD, and the allele G of rs4570625 might be associated with the severity of tic symptoms in boys. These polymorphisms might be susceptibility loci for TD in the Chinese Han population. Because of the confounding of co-existing attention deficit hyperactivity disorder (ADHD),these findings need to be confirmed by studies in much larger samples.

## Background

Tic disorder (TD) is a neuropsychiatric disorder characterized by involuntary tics (multiple motor and/or vocal tics). Tics wax and wane over time and are more intense in prepubertal boys. A large proportion of affected children reach a quiescent stage around early adolescence [1-3]. Recent epidemiological studies have changed the traditional perception that TD is rare, and demonstrate a prevalence rates >1000 per 100000 children [4-7]. Co morbidity of TD with attention deficit hyperactivity disorder (ADHD) and obsessive-compulsive disorder (OCD) is common [8].

The exact cause of TD is unknown. A heritable component is proposed to play a major role in the pathophysiology of TD, as shown by family studies, evaluations of monozygotic and dizygotic twins, and by follow-up investigations of at-risk children [9-12]. However, the susceptible genes or relevant DNA sequence variations that might be involved in the pathogenesis of TD have not been identified [13,14]. Abnormalities of neurotransmitter systems such as dopamine and serotonin (5-hydroxytryptamine, 5-HT) are also proposed to contribute to the symptoms of the disorder. Serotonergic projections from the dorsal raphe area have been shown to inhibit dopamine function in the striatum and cortex, where they inhibit the synaptic release of dopamine, and probably the synthesis of dopamine. Data from neurochemical studies have shown that generally decreased concentrations of 5-hydroxyindolacetic acid (5-HIAA) in cerebrospinal fluid (CSF) in TD patients could reflect the inadequate modulation of dopaminergic activity by serotonergic neurons [15]. Data from pharmacological studies also indicate the use of serotonin reuptake inhibitors (SRIs) is effective in tic reduction in some TD children [16,17]. Tryptophan hydroxylase (TPH) is a rate-limiting enzyme that catalyses the synthesis of 5-HT from tryptophan [18]. Until recently, it was thought that there was only one gene encoding TPH, referred to as tryptophan hydroxylase isoform (TPH1). However, TPH knockout studies in mice (mice TPH−/−) showed normal amounts of 5-HT in certain brain regions compared to their wild-type siblings (mice TPH+/+) [19]. Furthermore, TPH knockout mice exhibited no significant differences in 5-HT-related behaviors. This observation confirmed the hypothesis of the existence of a second TPH isoform (TPH2) [19]. The expression of both isoforms of TPH is partially exclusive. Several studies demonstrate that in humans TPH2 is exclusively expressed in serotonergic neurons of the brain, with the predominant expression in the Raphe nuclei of the midbrain, whereas TPH1 is mostly expressed in peripheral tissues [20]. TPH2 is located on chromosome 12q21.1; it spans a region of 93.5 kb and harbors 11 exons [20].

TPH2 is strategically placed to control 5-HT synthesis in the brain. For this reason, there has been great interest in identifying genetic variants that affect TPH2 enzymatic activity or the expression levels of the TPH2 gene. However, extensive DNA sequencing of the TPH2 gene has shown that the polymorphisms change the amino-acid sequence of the TPH2 protein were rare [21,22]. The research has, therefore, focused on identifying genetic variants that influence the TPH2 gene expression [23]. Some studies suggest that the human TPH2 gene includes several single nucleotide polymorphisms (SNPs) which include G to T base substitution in the transcriptional control region at position −703 (rs4570625). Recently, the functional relevance of rs4570625 in the transcriptional control region of the TPH2 gene was investigated by many groups [23-26]. In addition, the haplotype analysis of rs4570625 and rs4565946 by Mossner et al. found an association between TD and the G-C haplotype (and to a lesser extent, the T-C haplotype). This came from the association of the CC genotype and the C allele of the intronic SNP rs4565946 [27].

The current study was undertaken to investigate whether the TPH2 polymorphisms (SNP rs4570625 and rs4565945) act as susceptibility loci for TD in the Chinese Han population. We also investigated the possible association between these polymorphisms and severity scored with the Yale Global Tic Severity Scale (YGTSS) [28].

## Materials and methods

### Study subjects

The characteristics of the study participants are show in Table 1. A total of 149 children with TD, diagnosed according to the DSM-IV-TR criteria [29], were included in this study. These subjects were recruited from patients who were admitted to the Department of Neurological Medicine of Capital Institute of Pediatrics. Individuals with TD who also had coexisting diagnosis of OCD and anxiety disorder were excluded. All participants were from the Chinese Han population with a mean age of 8.90 years (range: 3 to 17 years). Thirty five children in this TD group had coexisting ADHD based on DSM-IV-TR criteria.

**Table 1:** Characteristics of study participants

**Characteristic**	**TD children**	**Controls**	***P***
	**(n = 149)**	**(n = 125)**	
**Sex (n,%)**			
Male	112(75.17)	84(67.20)	
Female	37(24.83)	41(32.80)	0.470^a^
**Age (Mean** ± SD**)**	8.90 ± 2.78	8.62 ± 2.61	0.220^b^
Male	8.92 ± 2.76	8.92 ± 2.94	1.000^b^
Female	8.84 ± 2.92	7.98 ± 1.99	0.129^b^
**Age of onset (year)**	6.67 ± 2.80		
**ADHD comorbidity (n,%)**			
TD only	114(76.51)		
TD + ADHD	35(23.49)		
**YGTSS (n,%)**			
low score subgroup	36(23.68)		
high score subgroup	116(76.32)		

Abbreviations:

*TD only*, children with only tics.

*TD + ADHD*, children with tics and ADHD.

a Chi-square test was used to calculate the p- values.

b *t* test was used to calculate the p- values.

An additional 125 ethnically matched subjects with a mean age of 8.62 years (range: 6 to 15 years) were selected from the general population in Beijing to serve as a control group. None of the control subjects or their family had a history of tics, ADHD, OCD, autism, anxiety disorder, or other psychiatric diagnoses.

The clinical assessment was made by neurologists in the Capital Institute of Pediatrics. The protocols for the study and the written consent were approved by the ethics committee of the Capital Institute of Pediatrics at Beijing, China (Approval ID: ey2010003).

### Assessment of symptom severity

The severity of tic symptoms was scored according to YGTSS [28], which included a clinician-completed rating scale and assessment of the overall degree of impairment. Symptom severity was assessed for five separate domains (number, frequency, intensity, complexity, and interference), with the defined maximum score of 50.

The mean severity of tic symptoms was 29.61 (range: 10 to 48). Based on the YGTSS, male TD children were further divided into two groups: a low score subgroup with lower levels of tic symptoms (YGTSS score: less than 25) and a high score subgroup with higher levels of tic symptoms (YGTSS score: 25 or more).

### DNA extraction and nucleotide sequences

Genomic DNA was extracted from frozen blood samples for genotyping using a blood and tissue DNA kit (Qiagen, Germany) according to manufacturer’s instructions. The concentration (A_260_ nm) and purity (A_260_/A_280_ ratio) of the DNA were determined with spectrophotometer.

Two single nucleotide polymorphisms (SNPs) found to represent common allelic variants of TPH2 in the general population were chosen for association analyses. Both SNPs were named in the present study according to ID numbers from the SNP database (http://www.ncbi.nlm.nih.gov/SNP/). The positions of the SNPs were taken from the Hapmap database (http://www.hapmap.org/). SNP rs4570625 was located in the putative transcriptional control region of TPH2, according to previously published data [23,30] and in accordance with our own assessments using an internet-based computational method (URL:http://thr.cit.nih.gov/molbio/proscan). The position of the SNP rs4570625 was -703bp upstream of the transcription start site. The SNP rs4565946 was located in intron 2 of TPH2.

### Genotyping

All samples were diluted to a DNA concentration of 20ng/μL. Two tagging SNPs (rs4565946 and rs4570625) across the TPH2 gene were selected for genotyping using data from the International Hapmap Project (2003). SNPs were genotyped using MassARRAY technology (Sequenom Inc., San Diego, CA, USA). The iPLEX™ assay was based on post-PCR single-base primer extension. The assay was performed according to manufacturer’s instructions. Forward, reverse and extension primers were designed using the Assay Design 3.0 software of Sequenom® (see Additional file 1: Table S1).

The iPLEX ™ reaction products were dispensed onto a 384-well SpectroChip (Sequenom Inc.), and they were processed and analyzed in a Compact Mass Spectrometer by Mass ARRAY Workstation 4.0 software (Sequenom Inc., San Diego, CA, USA).

To ensure consistency, 10% of samples were subjected to repeat genotyping assay. The accuracy of genotyping was validated by directly sequencing 10% of the samples. Reproducibility of the genotyping was high, with 100% concordance between the PCR and Sequnom® MassARRAY and direct sequencing methods.

Sequencing results were analyzed using Mutation Surveyor version 4.0.5 (Softgenetics, State College, Pennsylvania; http://www.softgenetics.com) for alignment and multiple comparisons.

### Statistical analysis

Statistical analyses were performed using SSPS version 16.0 for Windows (SPSS, Inc., Chicago, IL, USA). HAPLOVIEW 4.1 software was used to evaluate pair wise linkage disequilibrium (LD) and haplotype frequency between each tag SNP. Hardy-Weinberg equilibrium, genotype and allele frequencies of individual SNPs, and YGTSS and genotype groups at tic severity sections were analyzed using ANOVA or *t* tests for continuous variables or by Chi-square (χ^2^) or Fisher exact tests for categorical variables. Odds ratios (OR) with 95% confidence intervals (CI) were calculated to estimate the risks related to polymorphisms and TD. As the morbidity associated with TD is higher in males than in females [4], data from male subjects with TD were analyzed separately. Genotype and allele distributions of TPH2 SNPs in different ethnic populations were obtained from the Hapmap database (http://www.hapmap.org/). All p-values were 2-sided, and p-values <0.05 were considered statistically significant. For multiple comparisons of the genotype frequencies using the Bonferroni corrections, p-values <0.025 were considered significant.

## Results

There were no significant differences in age and gender distributions between the two groups (*P* > 0.05).

### Genotype and allele frequencies of TPH2 polymorphisms

Genotype information was successfully obtained from 274 participants (149 cases and 125 controls). Both SNPs were found to be in Hardy-Weinberg equilibrium (HWE) in controls (rs4565946: *P* = 0.49; rs4570625: *P* = 0.47). There was a significant deviation from the equilibrium at rs4565946 in TD children (rs4565946: *P* = 0.002; rs4570625: *P* = 1.00).

The distribution of allele and genotypes of the TPH2 polymorphisms in TD children and controls are shown in Table 2. There was a statistically significant difference in the genotype distributions of TPH2 rs4565946 between the TD children and normal controls. Homozygous TT was significantly higher in TD children than in normal controls (OR = 3.077, 95%CI, 1.273–7.437; *P* = 0.018 after Bonferroni correction). A significantly increased frequency of the TT genotype was also noticed in male TD children (OR = 3.228, 95%CI, 1.153–9.040; *P* = 0.040 after Bonferroni correction). There was no significant difference in allele frequency between male TD children and male controls after Bonferroni correction (*P* > 0.025) (Table 3).

**Table 2:** Genotype and Allele frequencies of TPH2 gene polymorphisms between children with TD and normal controls

**Polymorphisms**	**Number and frequency (%)**		***χ***^**2**^	***P***^**a**^	**OR [95% CI]**
	**Normal controls**	**TD children**			
**TPH2 rs4565946**	**125**	**149**			
CC	65 (52.00)	78 (52.35)			1.00
CT	53 (42.40)	48 (32.21)`	1.168	0.28	0.755 [0.453–1.258]
TT	7 (5.60)	23 (15.44)	4.994	0.025	2.738 [1.105–6.788]
CC + CT	118 (94.40)	126 (84.56)			1.00
TT	7 (5.60)	23 (15.44)	6.745	0.009	3.077 [1.273–7.437]
C	183 (73.20)	204 (68.46)			1.00
T	67 (26.80)	94 (31.54)	1.474	0.225	1.259 [0.868–1.825]
**TPH2 rs4570625**	**125**	**149**			
TT	44 (35.20)	37 (24.83)			1.00
GT	57 (45.60)	75 (50.34)	2.498	0.114	1.565 [0.897–2.730]
GG	24 (19.20)	37 (24.83)	3.127	0.077	1.833 [0.934–3.599]
TT + GT	101 (80.80)	112 (75.17)			1.00
GG	24 (19.20)	37 (24.83)	1.246	0.264	1.390 [0.779–2.483]
T	145 (58.00)	149 (50.00)			1.00
G	105 (42.00)	149 (50.00)	3.499	0.061	1.381 [0.984–1.937]

Abbreviations: TPH2 = tryptophan hydroxylase2.

^a^ Chi-square test was used to calculate the p-values.

**Table 3:** Genotype and allele frequencies of TPH2 gene polymorphisms between children with TD and normal controls in the male subgroup

**Polymorphisms**	**Number and frequency (%)**		***χ***^**2**^	***P***^**a**^	**OR [95% CI]**
	**Male normal controls**	**Male TD children**			
**TPH2 rs4565946**	**84**	**112**			
CC	46 (54.76)	58 (51.79)			
CT	33 (39.29)	35 (31.25)	0.306	0.580	0.841 [0.456–1.553]
TT	5 (5.95)	19 (16.96)	4.454	0.035	3.014 [1.046–8.685]
CC + CT	79 (94.05)	93 (83.04)			
TT	5 (5.95)	19 (16.96)	5.417	0.020	3.228 [1.153–9.040]
C	125 (74.40)	151 (67.41)			
T	43 (25.60)	73 (32.59)	2.254	0.133	1.405 [0.900–2.193]
**TPH2 rs4570625**	**84**	**112**			
TT	29 (34.52)	29 (25.89)			
GT	40 (47.62)	50 (44.64)	0.437	0.508	1.250 [0.645–2.423]
GG	15 (17.86)	33 (29.46)	3.803	0.051	2.200 [0.990–4.888]
TT + GT	69 (82.14)	79 (70.54)			
GG	15 (17.86)	33 (29.46)	3.497	0.061	1.922 [0.963–3.833]
T	98 (58.33)	108 (48.21)			
G	70 (41.67)	116 (51.79)	3.942	0.047	1.504 [1.005- 2.251]

Abbreviations: *TPH2*, tryptophan hydroxylase2.

^a^Chi-square test was used to calculate the *p-* values.

There were no differences in allele and genotype frequencies of SNP rs4570625, between TD children and normal controls after Bonferroni correction (*P* > 0.025). TD children had nominally higher frequencies of G allele than the normal controls in males (OR = 1.504, 95%CI, 1.005–2.251; *P* = 0.047) (Table 3), but the difference was not statistically significant after Bonferroni correction (*P* = 0.047 and *P* = 0.094).

No differences between TD children and normal controls in the female subgroups were identified for the two polymorphisms (shown in Additional file 1: Table S2).

### Allelic and genotypic association analyses of TPH2 polymorphisms and severity of TD in male children

ANOVA analyses did not show any genetic effect at either SNP in the patient group (shown in Additional file 1: Table S3).

Differences in symptom severity among genotypes were found between the high score subgroup of TD children, and normal controls in males (Table 4). SNP rs4570625 showed a significant increase of G-allele in the high score subgroup (OR = 1.684, 95%CI, 1.097–2.583; *P* = 0.034 after Bonferroni correction). A marginal significant *p*-values (*P* = 0.045) was observed for GG versus GT plus TT genotypes in the high score subgroup versus male controls, but this was not significant after Bonferroni correction (*P* = 0.045 and *P* = 0.090). However, the high score subgroup had significantly higher frequencies of the SNP rs4546946 TT genotype than did normal controls in male children (*P* = 0.044 after Bonferroni correction).

**Table 4:** Allelic and genotypic association analyses of TPH2 polymorphisms in low score subgroup and high score subgroup versus normal controls among the male children

**Polymorphisms**	**Number and frequency (%)**		
	**Male normal controls**	**Low score subgroup**	**High score subgroup**
**rs4565946**	**84**	**25**	**87**
CC	46 (54.76)	15 (60.00)	43 (49.43)
CT	33 (39.29)	6 (24.00)	29 (33.33)
TT	5 (5.95)	4 (16.00)	15 (17.24)
*χ*^2^		3.708	5.308
*P*^a^		0.157	0.070
CC + CT	79 (94.05)	21 (84.00)	72 (82.76)
TT	5 (5.95)	4 (16.00)	15 (17.24
*χ*^2^		2.568	5.273
*P*^b^		0.206	0.022
OR [95%CI]		3.010 [0.742–12.205]	3.292 [1.139–9.513]
C	125 (74.40)	36 (72.00)	115 (66.09)
T	43 (25.60)	14 (28.00)	59 (33.91)
*χ*^2^		0.115	2.822
*P*^b^		0.734	0.093
OR [95%CI]		1.130 [0.557–2.295]	1.491 [0.934–2.380]
**rs4570625**	**84**	**25**	**87**
TT	29 (34.52)	10 (40.00)	19 (21.84)
GT	40 (47.62)	9 (36.00)	41 (47.13)
GG	15 (17.86)	6 (24.00)	27 (31.03)
*χ*^2^		1.117	5.473
*P*^a^		0.572	0.065
TT + GT	69 (82.14)	19 (76.00)	60 (68.97)
GG	15 (17.86)	6 (24.00)	27 (31.03)
*χ*^2^		0.467	4.005
*P*^b^		0.494	0.045
OR [95%CI]		1.453 [0.496–4.253]	2.070 [1.008–4.252]
T	98 (58.33)	29 (58.00)	79 (45.40)
G	70 (41.67)	21 (42.00)	95 (54.60)
*χ*^2^		0.002	5.724
*P*^b^		0.967	0.017
OR [95%CI]		1.014 [0.535–1.922]	1.684 [1.097–2.583]

^a^ Pearson chi square test was used to calculate the p-values.

^b^Chi-square test was used to calculate the p-values. Fisher exact test was used when the sample size was <5.

### Haplotype analyses and linkage disequilibrium with TPH2 rs4570625 and rs4565946

The two tag SNPs showed a D’ value of 0.933 (r^2^ = 0.429) suggesting strong linkage disequilibrium (LD). A two-marker haplotype analysis was performed across the TPH2 gene with both SNPs. As shown in Table 5, there was a trend with C-G and T-G haplotypes increase risk to TD (OR = 1.411, 95%CI, 0.732–2.718; OR = 1.356, 95%CI, 0.781–2.356; respectively), but the differences were not significant (*P* > 0.05).

**Table 5:** Haplotype frequency between children with TD and normal controls

**Haplotypes**^**a**^	**Number and frequency (%)**		***χ***^**2**^	***P***	**OR [95% CI]**
	**TD children**^**b**^	**Normal controls**^**b**^			
C-T	74(49.60)	72(57.53)			
T-G	47(31.15)	33(26.33)	1.172	0.279	1.356 [0.781–2.356]
C-G	28(18.85)	20(15.67)	1.063	0.302	1.411 [0.732–2.718]

^a^Haplotypes were constructed using rs4565946 and rs4570625 single-nucleotide polymorphisms, from left to right. No T-T haplotype was observed.

^b^ Percentages may not equal 100 because of rounding.

Table 6 shows the frequencies of the estimated 2-marker haplotypes among TD children and normal controls in males. There was also a trend that C-G and T-G haplotypes increase risk to TD in males (OR = 1.426, 95%CI, 0.658–3.091; OR = 1.556, 95%CI, 0.802–3.018; respectively), but the differences were not significant (*P* > 0.05).

**Table 6:** Haplotype Frequency between children with TD and normal controls among the male children

**Haplotypes**^**a**^	**Number and frequency (%)**		***χ***^**2**^	***P***	**OR [95%CI]**
	**Male TD children**	**Male normal controls**^**b**^			
C-T	54(48.21)	49(57.63)			
T-G	36(32.59)	21(24.89)	1.717	0.190	1.556 [0.802–3.018]
C-G	22(19.2)	14(16.77)	0.812	0.368	1.426 [0.658–3.091]

^a^Haplotypes were constructed using rs4565946 and rs4570625 single-nucleotide polymorphisms, from left to right. No T-T haplotype was observed.

^b^ Percentages may not equal 100 because of rounding.

We also compared our results of the two sites with Hapmap database (http://hapmap.ncbi.nlm.nih.gov/), and found that genotype and allele distributions were significantly different in different ethnic populations, especially in European populations (*P* < 0.05) (Table 7).

**Table 7:** Genotype and allele frequencies of the TPH2 SNP rs4565946 in normal population of different races

**Polymorphism**	**Number and frequency (%)**							
	**Chinese (the paper)**	**CHB (H)**	**ASW (A)**	**CEU (C)**	**GIH (G)**	**JPT (J)**	**MEX (M)**	**TSI (T)**
**TPH2 rs4565946**								
**N**	**125**	**137**	**57**	**113**	**101**	**113**	**58**	**101**
CC	65(52.0)	81 (46.6)	44 (77.2)	30 (26.5)	40 (39.6)	49 (43.4)	29 (50.0)	34 (33.7)
CT	53 (42.4)	63 (46.0)	10 (17.5)	51 (45.1)	47 (46.5)	51 (45.1)	27 (46.6)	43 (42.6)
TT	7 (5.6)	8 (5.8)	3 (5.3)	32 (28.3)	14 (13.9)	13 (11.5)	2 (3.4)	24 (23.8)
*P*^a^		0.975	0.004	<0.001	0.046	0.175	0.756	<0.001
C	183 (73.2)	195 (71.2)	98 (86.0)	111 (49.1)	127 (62.9)	149 (65.9)	85 (73.3)	111(55.0)
T	67 (26.8)	79 (28.8)	16 (14.0)	115 (50.9)	75 (37.1)	77 (34.1)	31 (26.7)	91 (45.0)
*P*^b^		0.604	0.007	<0.001	0.019	0.085	0.988	<0.001
**TPH2 rs4570625**								
**N**	**125**	**136**	**57**	**113**	**101**	**113**	**57**	**102**
GG	24 (19.2)	30 (22.1)	26 (45.6)	73 (64.6)	63 (62.4)	28 (24.8)	25 (43.9)	60 (58.8)
GT	57 (45.6)	71 (52.2)	25 (43.9)	33 (29.2)	32 (31.7)	58 (51.3)	27 (47.4)	33 (32.4)
TT	44 (35.2)	35 (25.7)	6 (10.5)	7 (6.2)	6 (5.9)	27 (23.9)	5 (8.8)	9 (8.8)
*P*^a^		0.251	<0.001	<0.001	<0.001	0.150	<0.001	<0.001
G	105 (42.0)	131 (48.2)	77 (67.5)	179 (79.2)	158 (78.2)	114 (50.4)	77 (67.5)	153 (75.0)
T	145 (58.0)	141 (51.8)	37 (32.5)	47 (20.8)	44 (21.8)	112 (49.6)	37 (32.5)	51 (25.0)
*P*^b^		0.158	<0.001	<0.001	<0.001	0.065	<0.001	<0.001

Abbreviations: *TPH2*, tryptophan hydroxylase2.

*CHB (H)*, Han Chinese in Beijing, China; *ASW (A)*, African ancestry in Southwest USA; *CEU (C)*, Utah residents with Northern and Western European ancestry from the CEPH collection; *GIH (G)*, Gujarati Indians in Houston, Texas; *JPT (J)*, Japanese in Tokyo, Japan; *MEX (M)*, Mexican ancestry in Los Angeles, California; *TSI (T)*, Tuscan in Italy;

^a^ Pearson Chi square test was used to calculate the p-values.

^b^ Chi square test was used to calculate the p-values. Continuity Correction was used when the minimum expected was <5.

### Transcription factor binding sites

**Figure 1:**
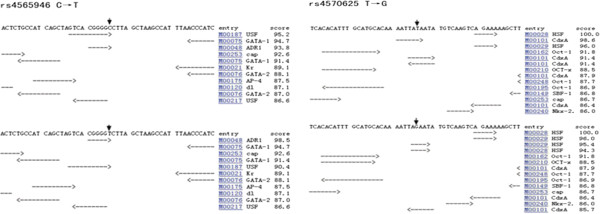
**Structure of the predicted transcription factor binding sites. A**: Transcription factor binding sites for function prediction in major allele. **B**: Transcription factor binding sites for function prediction in minor allele.

Based on transcription factor binding sites prediction (http://www.cbrc.jp/research/db/ TFSEARCH.html; Figure 1), promoter rs4570625 (G > T) affects the binding ability to the CdxA transcription factor. Intron 2 rs4565946 (C > T) demonstrated no change in ability to combine with the transcription factor (Figure 1).

## Discussion

TD is thought to be caused by a complex interaction of environmental and biological factors, with multiple genes acting at distinct neural circuits, such as the brain serotonin system. Several studies have investigated an association between the TPH2 gene and other psychiatric disorders, but only one study to date has demonstrated an association between TPH2 and Tourette’s syndrome (TS) [27]. Mossner et al. investigated an association between two SNPs (rs4570625 and rs4565946) of the TPH2 gene and TS in German samples and showed that the C allele and CC genotype of rs4565946 was associated with TS [27]. This finding is not in line with our results, which suggest that the C allele is the major allele for TPH2 rs4565946 in Chinese subjects, indicating that there are ethnic differences in comparison with European, Indian, and other populations (Table 7). Our results do, however, suggest that the T allele of rs4565946 may be the risk allele in the Chinese Han population, and that individuals with T allele may be more susceptible to TD than those with C allele. We, therefore, propose that the TT genotype might produce different effects on TD in subjects in different ethnic groups.

It has been suggested that alteration in TPH2 gene expression might result in central serotonergic system dysfunction, which may in turn lead to abnormality of behavior and increased susceptibility to psychiatric disorders such as TS [27]. Chen et al. demonstrated that the expression of human TPH2 is regulated by both the 5’-UTR and common polymorphisms in the 5’ regulatory region of TPH2 [26]. These researchers also suggest that promoter rs4570625 (−703 G/T) is likely to influence the transcription rate of TPH2, a proposal supported by transcription factor binding sites for function prediction (http://www.cbrc.jp/research/db/TFSEARCH.html). In our study, TD children had significantly higher frequencies of the rs4570625 G allele than the normal controls in males, suggesting that the G allele might increase the risk of TD in the Chinese Han population.

Additional evidence has shown that the T allele of rs4570625 is related to the low expression of TPH2 and increased activity in the prefrontal brain [31,32]. James F et al. demonstrated that levels of tryptophan in cerebrospinal fluid were inversely associated with tic severity in TS subjects [33]. In our studies, we found an increase in G-allele in high score subgroup of male TD children. For rs4570625, the allele G type might affect the transcription factor binding ability (CdxA and HSF); therefore, the allele G type can change the transcription regulation of the *TPH2* gene. The results indicated that the allele G of rs4570625 might have relationship with the severity of tic symptoms among male TS children in the Chinese Han population.

A recent study provided evidence that promoter rs4570625 polymorphism was not associated with down-regulated expression of TPH2 [30]. Whether SNP rs4570625 is a direct disease-causing polymorphism, therefore, remains a subject of further study.

To the best of our knowledge, no previous studies have investigated whether the intronic variant (SNP rs4565946) has any functional effects. In our study, the intronic polymorphism rs4565946 resulted in strong linkage disequilibrium (D’ = 0.933, r^2^ = 0.429) with a functional polymorphism of rs4570625. Considering the association of TD with the T-G and C-G haplotypes, and given results for each SNP and the observation that the C allele located in intron 2 is in linkage disequilibrium with the functional T allele of the promoter SNP, it is possible that the intronic variant may also be functionally important in the development of TD.

There are a number of limitations to the present study. Firstly, the relatively small sample size and only two SNPs were studied, may have reduced the power to detect the main effects of (serotonin-related) TPH2 gene polymorphisms and limit the generalization of our results to the population as a whole. Secondly, nearly 23.5% of TD children in our study had ADHD, therefore, our results could be confounded by co-existing ADHD. Thirdly, no information was available about the medications and behavioral therapeutic interventions of the TD patients at the recruitment. For these reasons our data should be interpreted cautiously.

## Conclusions

This is the first study to assess whether polymorphic variants of TPH2 are associated with TD in the Chinese Han population. Our results clearly link TPH2 variations to the pathogenesis of TD and further support the etiological relevance of 5-HT signaling in TD.

## Abbreviations

ADHD: Attention deficit hyperactivity disorder; HWE: Hardy-weinberg equilibrium; LD: Linkage disequilibrium; OCD: Obsessive-compulsive disorder; OR: Odds ratio; SNPs: Single nucleotide polymorphisms; TD: Tic disorder; TPH2: Tryptophan hydroxylase-2; YGTSS: Yale global tic severity scale.

## Competing interests

The authors declare that they have no competing interests.

## Authors’ contributions

PZ participated in experimental studies and statistical analysis, and drafted the manuscript. E-zL and J-hW participated in the design of the study and performed the data analysis. L-wW participated in providing both clinical information and samples for the study. X-dC provided the experiment guidance. All authors read and approved the final manuscript.

## Supplementary Material

Additional file 1 Table S1 The iPLEX primers of two SNP sites of TPH2 gene. **Table S2.** Genotype and allele frequencies of TPH2 gene polymorphisms between children with TD and normal controls in the female subgroups. **Table S3.** Genetic association analyses of TPH2 polymorphisms. Click here for file
