# Supplementary material for: Involvement of tryptophan hydroxylase 2 gene polymorphisms in susceptibility to tic disorder in Chinese Han population
Source: Behav Brain Funct. 2013 Jan 29;9:6. doi: 10.1186/1744-9081-9-6 (PMC3573945; doi:10.1186/1744-9081-9-6)
Supplement: Additional file 1 Table S1 — The iPLEX primers of two SNP sites of TPH2 gene. Table S2. Genotype and allele frequencies of TPH2 gene polymorphisms between children with TD and normal controls in the female subgroups. Table S3. Genetic association analyses of TPH2 polymorphisms. [file 1744-9081-9-6-S1.doc]

**Additional material**

**Supplementary Table 1**-The iPLEX primers of two SNP sites of TPH2 gene

| **SNP Sites** | **Sequences of primers: 5’→3’** | **Length of PCR products** |
| --- | --- | --- |
| Promoter  −703G/T  rs4570625 | 4570625 Forward primer  ACGTTGGATGACTCACACATTTGCATGCAC | 96bp |
|  | 4570625 Reverse primer:  ACGTTGGATGTGACCAACTCCATTTTATG |  |
|  | 4570625UEP:AAGCTTTTTCTGACTTGACATATT | 1bp |
| Intron 2  rs4565946 | 4565946 Forward primer:  ACGTTGGATGGTCCAGATGGGTTAAATGGC | 100bp |
|  | 4565946 Reverse primer:  ACGTTGGATGAAGGAGTTAGCAGCCCTGAG |  |
|  | 4565946UEP:ATCAGCTAGTCACGGGG | 1bp |

**Supplementary Table 2**-Genotype and allele frequencies of TPH2 gene polymorphisms between children with TD and normal controls in the female subgroups

| **Polymorphisms** | **Number and frequency (%)** | | **χ^2^** | **P^a^** | **OR [95% CI]** |
| --- | --- | --- | --- | --- | --- |
|  | **female normal controls** | **female TD children** |  |  |  |
| **TPH2 rs4565946** | **41** | **37** |  |  |  |
| CC | 19 (46.34) | 20 (54.05) |  |  |  |
| CT | 20 (48.78) | 13 (35.14) | 1.018 | 0.313 | 0.618 [0.241-1.579] |
| TT | 2 (4.88) | 4 (10.81) | 0.495 | 0.670 | 1.900 [0.311-11.607] |
| C | 58 (70.73) | 53 (71.62) |  |  |  |
| T | 24 (29.27) | 21 (28.38) | 0.015 | 0.902 | 0.958 [0.478-1.917] |
| **TPH2 rs4570625** | **41** | **37** |  |  |  |
| TT | 15 (36.59) | 8 (21.62) |  |  |  |
| GT | 17 (41.47) | 25 (67.57) | 3.640 | 0.056 | 2.757 [0.959-7.930] |
| GG | 9 (21.95) | 4 (10.81) | 0.060 | 0.806 | 0.833 [0.194-3.578] |
| T | 47 (57.32) | 41 (55.41) |  |  |  |
| G | 35 (42.68) | 33 (44.59) | 0.058 | 0.810 | 1.081 [0.574- 2.037] |

^a^ Chi-square test was used to calculate the p-values. Fisher exact test was used when the sample size was <5.

**Supplementary Table 3**-Genetic association analyses of TPH2 polymorphisms

| TPH2 | N | Mean±SD | F | P**a** |
| --- | --- | --- | --- | --- |
| rs4565946 |  |  |  |  |
| CC | 78 | 29.71±7.21 |  |  |
| CT | 48 | 30.31±7.82 |  |  |
| TT | 23 | 29.52±8.47 | 0.12 | 0.88 |
| rs4570625 |  |  |  |  |
| TT | 37 | 28.97±7.76 |  |  |
| GT | 75 | 29.93±7.37 |  |  |
| GG | 37 | 30.65±7.88 | 0.46 | 0.64 |

^a^ ANOVA analyses were used to calculate the p- values.
